# *Sclerotinia* spp. causing root rot of *Panax ginseng* in Northeast China and its potential biocontrol by *Bacillus amyloliquefaciens* FS6

**DOI:** 10.1128/spectrum.00470-25

**Published:** 2025-09-03

**Authors:** Shi Feng, Qihang Li, Xue Wang, Mingyuan Hu, Lina Yang, Changqing Chen, Baohui Lu, Jie Gao

**Affiliations:** 1 College of Plant Protection, Jilin Agricultural University 85112, Changchun, China; 2 State-Local Joint Engineering Research Center of Ginseng Breeding and Application, Changchun, China; 3 Key Laboratory of Integrated Pest Management on Crops in Northeast Ministry of Agriculture and Rural Affairs, Gongzhuling, China; USDA-ARS San Joaquin Valley Agricultural Sciences Center, Parlier, California, USA

**Keywords:** *Sclerotinia* root rot, *Panax ginseng*, pathogen isolation, phylogenetic analysis, biological control, control efficiency

## Abstract

Sclerotinia root rot is one of the most destructive soilborne diseases in ginseng (Asian ginseng) production regions in China. To identify the causal pathogens, 409 *Sclerotinia* isolates were recovered from ginseng plants at 11 plantations in Jilin, Heilongjiang, and Liaoning Provinces in China from 2018 to 2021. Based on morphological characteristics, sequence analyses of the internal transcribed spacer region and the beta-tubulin gene, as well as a phylogenetic analysis, the isolates were identified as *S. nivalis* (*n* = 268), *S. ginseng* (*n* = 114), and *S. sclerotiorum* (*n* = 27). *S. nivalis* and *S. sclerotiorum* were found on fields growing *Panax ginseng* for the first time in China. *Bacillus amyloliquefaciens* FS6 (CGMCC 9538) exerted significant antifungal effects on all three *Sclerotinia* spp. by inhibiting *in vitro* mycelial growth by 85%, resulting in deformed and broken hyphae and dissolving sclerotia; on average, >73% inhibition (sclerotia formation) was achieved by FS6. Notably, the germination of sclerotia was inhibited for up to 20 days post-treatment in the fermentation broth (FB) of FS6 at concentrations of 4 × 10^6^ to 4 × 10^8^ CFU·mL^−1^. Using those FS6 FB concentrations *in vitro* resulted in control efficacy of 43.48% to 100.00% against Sclerotinia root rot. Field trials in 2021 and 2022 showed that 10^7^ to 10^8^ CFU·mL^−1^ FS6 FB sprayed on ginseng leaves three times every 7 days exhibited a promising control effect against *S. nivalis*, with a control efficiency ranging from 53.00% to 73.51% 15 days after the third application. This study clarified that Sclerotinia root rot affecting Asian ginseng in Northeast China is caused by three species of *Sclerotinia*, for which *B. amyloliquefaciens* FS6 is a promising biocontrol agent.

**IMPORTANCE:**

Sclerotinia root rot typically causes substantial losses in ginseng yield. This study aims to identify the types of pathogens that cause ginseng sclerotinia rot and their distribution in various production areas. This study is the first to report *S. nivalis* as a dominant species causing sclerotinia root rot in Asian ginseng cultivated in China. The most effective method for the prevention and control of sclerotinia root rot is biological control. Our findings suggest that *B. amyloliquefaciens* FS6 could be a promising way to control sclerotinia disease prevalent in commercial ginseng plantations in China.

## INTRODUCTION

Asian ginseng (*Panax ginseng* C. A. Meyer) is a perennial herbaceous medicinal plant in the Araliaceae family that is mainly cultivated in Northeast China, North Korea, South Korea, Japan, and Eastern Russia. Asian ginseng has extremely important medicinal and commercial value (1). However, cultivated ginseng is extremely vulnerable to a variety of pathogens (2, 3). Common diseases on ginseng include Alternaria blight (*Alternaria* spp.) (4), gray mold (*Botrytis* spp.) (5), anthracnose (*Colletotrichum* spp.) (6), damping-off (*Globisporangium debaryanum*, *Globisporangium ultimum*, and *Rhizoctonia solani*) (7), and root rot (*Fusarium* spp., *Sclerotinia ginseng*, and *Ilyonectria* spp.) (8–10). Among these fungal diseases, those caused by *Sclerotinia* spp. are some of the most destructive soil-borne diseases worldwide, limiting ginseng production (11). The disease significantly reduces the yield and quality of ginseng root and impairs storage after harvesting. The pathogen was first isolated from the root of American ginseng in 1912 and identified as *Sclerotinia* sp (12). Later, it was identified as *S. sclerotiorum* by Whetzel et al. (3). In Korea, Nakata et al. (13) reported a fungus causing root rot on *P. ginseng,* which differed morphologically from *Sclerotinia sclerotiorum*. Decades later, Chen et al. (14), Qi et al. (15), and Wang et al. (16) found Sclerotinia root rot on Asian ginseng in China and identified the pathogen as *Sclerotinia ginseng. S. ginseng* was first reported as a causal agent of Asian ginseng root rot in Korea (17). *S. nivalis* was reported as a disease pathogen causing white rot on Asian ginseng in Korea in 2013 (9), on *Panax quinquefolius* in Taibai County, Shaanxi Province, China, 2021 (18) and roots of ginseng storage in Tonghua city of China in 2022 (19). Guan et al. (20) reported *S. sclerotium* causing root rot on *P. quinquefolius* in Fusong County, Jilin Province, China, in 2022. *S. nivalis* has not been reported on naturally growing ginseng in the field.

The *Sclerotinia* mycelium forms a unique structure called the sclerotia, which plays a key role as the source of primary infection in the disease cycle of Sclerotinia root rot (17). It is difficult to control this disease once it occurs because of the long-term survival of sclerotia in soil and its wide range of plant hosts. Traditional methods, such as crop rotation and applying resistant cultivars, are not completely effective. Chemical control is fast and efficient but is problematic because of ensuing residues and environmental pollution. Biological control, which is environmentally friendly and does not lead to resistance, has emerged as a trend in disease control (21).

*Bacillus* species are widely used to control plant diseases. In recent years, many researchers have isolated and identified *Bacillus* strains with antagonistic effects against pathogenic fungi from different plants and successfully applied them in production settings (22–24). Few studies are available on the control of root rot disease on ginseng used by *Bacillus* spp. However, only the isolation of biocontrol strains and evaluations of their inhibitory effects against *Cylindrocladium destructans*, *Rhizoctonia solani*, *Sclerotinia nivalis*, and *Fusarium solani* were conducted, with no further in-depth research carried out (25). *Bacillus amyloliquefaciens* FS6 was isolated from the *P. ginseng* rhizosphere soil by our laboratory and has significant effects against *P. ginseng* pathogens, such as *Botrytis cinerea* (26), *Alternaria* spp. (26), *Colletotrichum* spp. (6), and *Fusarium solani* (27). The FS6 strain shows excellent biocontrol efficacy on diseases afflicting ginseng seedlings caused by several agents, as well as gray mold caused by *B. cinerea* under field conditions (26). *B. amyloliquefaciens* FS6 readily colonized in ginseng hosts and its rhizosphere soil, which transmitted stably from soil to all parts of ginseng plants in greenhouse and field environments (26, 28). Our previous studies have demonstrated that the application of *B. amyloliquefaciens* FS6 to ginseng cultivation soil restored microbial equilibrium within 60 days, indicating its soil-environmentally benign properties. While studies on its effects on other crops and disease control efficacy are currently underway, no peer-reviewed publications have been documented to date. Whether *B. amyloliquefaciens* FS6 is effective against *Sclerotinia* spp. or is capable of inducing resistance on *P. ginseng* has not yet been reported.

*Sclerotinia* species were initially classified based on their morphological characteristics and host range, but recent taxonomic research has shown that multi-locus sequence analysis is the most effective way to study the taxonomy and genetic diversity of *Sclerotinia* (28). Nevertheless, systematic research on ginseng root rot in China caused by *Sclerotinia* is lacking. Hence, the objectives of this study were to identify the pathogenic *Sclerotinia* species capable of causing ginseng root rot based on morphological and molecular characteristics and to explore the biocontrol potential of *B. amyloliquefaciens* FS6 against this disease. Our findings will enhance the understanding of *B. amyloliquefaciens* FS6 and provide new strains for strategic biocontrol of sclerotinia root rot in ginseng.

## MATERIALS AND METHODS

### Sample collection

Sclerotinia root rot on ginseng was investigated from 2018 to 2021. According to the incidence of the disease, 2 to 65 samples were collected from each region by digging the diseased ginseng roots with a small iron shovel in the main commercial planting areas of Jilin, Liaoning, and Heilongjiang provinces. Information collected on the field samples is shown in Table 1.

**TABLE 1:** Isolate characteristics of three species of *Sclerotinia*

Species	Isolate	Substrate	Locality	Total isolates	Pathogenicity testing strains	Date	Morphotype
*S. nivalis*	YC5-YC7	Roots	Ji‘an, Tonghua, and Jilin, China	3	YC5	2018.09	The mycelia were white at the initial stage on PDA plates, dissolving-like and appearing white-yellowish at the late stage. Sclerotia were produced after 15 days and were arranged in two circles in a concentric ring. These sclerotia were spherical, hemispherical, and kidney-shaped and were easily separated from the culture plate. The number of sclerotia was moderate. After 20 days of culture, 160–180 sclerotia were produced in a 9 cm Petri dish, and their average size was 22.17 ± 1.64 mm ×22.90 ± 1.83 mm.
*S. nivalis*	8-1 to 71-1	Roots	Fusong, Baishan, Jilin, China	63	9-1, 11-1, 30-1	2019.05	
*S. nivalis*	CS79-1 to CS93-1	Roots	Fusong, Baishan, Jilin, China	15	CS93-1	2019.05	
*S. nivalis*	CS79-2 to CS93-2	Roots	Fusong, Baishan, Jilin, China	15		2019.05	
*S. nivalis*	SJH1 to SJH4	Roots	Fusong, Baishan, Jilin, China	4	SJH4	2019.06	
*S. nivalis*	WQG5-1 to WQG41-1	Roots	Wangqing, Yanbian, Jilin, China	37		2019.09	
*S. nivalis*	WQG5-2 to WQG41-2	Roots	Wangqing, Yanbian, Jilin, China	37		2019.09	
*S. nivalis*	TWG1 to TWG5	Roots	Ji’an, Tonghua, Jilin, China	5	TWG2	2019.09	
*S. nivalis*	LHG1 to LHG3	Roots	Liuhe, Tonghua, Jilin, China	3		2019.10	
*S. nivalis*	CBG1	Roots	Changbai, Baishan, Jilin, China	1		2020.06	
*S. nivalis*	CBJ1 to CBJ4	Stems	Changbai, Baishan, Jilin, China	4	CBJ1, CBJ2	2020.06	
*S. nivalis*	CBJ1-2 to CBJ4-2	Stems	Changbai, Baishan, Jilin, China	4		2020.06	
*S. nivalis*	BQ1-1 to BQ4-1	Stems	Baoqing, Shuangyashan, Heilongjiang, China	4		2020.06	
*S. nivalis*	BQ1-2 to BQ4-2	Stems	Baoqing, Shuangyashan, Heilongjiang, China	4		2020.06	
*S. nivalis*	BQ1-3 to BQ4-3	Stems	Baoqing, Shuangyashan, Heilongjiang, China	4	BQ1-3, BQ3-3	2020.06	
*S. nivalis*	JAG1-1 to JAG2-1	Roots	Ji’an, Tonghua, Jilin, China	2	JAG2-1	2020.06	
*S. nivalis*	JAG1-2 to JAG2-2	Roots	Ji’an, Tonghua, Jilin, China	2		2020.06	
*S. nivalis*	JAY1-1 to JAY4-1	Stems	Ji’an, Tonghua, Jilin, China	4		2020.06	
*S. nivalis*	JAY1-3 to JAY4-3	Stems	Ji’an, Tonghua, Jilin, China	4		2020.06	
*S. nivalis*	LHG4-1	Roots	Liuhe, Tonghua, Jilin, China	1	LHG4-1	2020.06	
*S. nivalis*	JY1-JY2	Stems	Jingyu, Baishan, Jilin, China	2		2020.06	
*S. nivalis*	HRG1 to HRG3	Roots	Huanren, Benxi, Liaoning, China	3	HRG2	2020.06	
*S. nivalis*	KD616 to KD618	Roots	Kuandian, Dandong, Liaoning, China	3	KD616, KD618	2020.06	
*S. nivalis*	KD2-1 to KD6-1	Roots	Kuandian, Dandong, Liaoning, China	5		2020.06	
*S. nivalis*	NMW1-1 to 4-1	Stems	Kuandian, Dandong, Liaoning, China	4	NMW3-1	2020.06.	
*S. nivalis*	NMW1-2 to 4-2	Stems	Kuandian, Dandong, Liaoning, China	4		2020.06	
*S. nivalis*	QY1-1 to QY1-3	Roots	Fusong, Baishan, Jilin, China	3	QY1-3	2020.06	
*S. nivalis*	QYJ2-1 to QYJ2-3	Roots	Fusong, Baishan, Jilin, China	3	QYJ2-1	2020.06	
*S. nivalis*	QYJ3-1 to QYJ3-3	Stems	Fusong, Baishan, Jilin, China	3		2020.06	
*S. nivalis*	YBL1 to YBL4	Roots	Yabuli, Haerbin, Heilongjiang, China	4		2020.09	
*S. nivalis*	SJH4-2 to SJH5-2	Roots	Fusong, Baishan, Jilin, China	6	SJH5-2	2019.06	
*S. nivalis*	TWG1 to TWG5	Roots	Tonghua, Tonghua, Jilin, China	5		2019.06	
*S. nivalis*	LSH1 to LSH3	Roots	Fusong, Baishan, Jilin, China	3		2020.06	
*S. ginseng*	TH1-TH2	Roots	Tonghua, Tonghua, Jilin, China	2	TH1, TH2	2018.05	The mycelia appeared white and dissolving-like at the earlier stage on PDA plates, but then turned gray-black during the late stage, and sclerotia were produced after 15–20 days. These sclerotia were arranged in one spherical and hemispherical ring, of which some were irregular in shape and lay close to the surface of the plate. A moderate number of sclerotia was produced: 132–161 on one 9-cm Petri dish after 20 days of culture on PDA. The sclerotia were medium-sized, averaging 22.53 ± 1.06 mm × 24.63 ± 1.10 mm.
*S. ginseng*	YC1-YC4	Roots	Ji’an, Tonghua, Jilin, China	4	YC1, YC2, and YC3	2018.09	
*S. ginseng*	JH2	Roots	Jingyue, Changchun, Jilin, China	1	JH2	2018.05	
*S. ginseng*	NDYY1 to NDYY6	Roots	Jingyue, Changchun, Jilin, China	6		2018.05	
*S. ginseng*	AT1 to AT5	Roots	Antu, Yanbian, Jilin, China	5	AT4	2018.03	
*S. ginseng*	WQJ1-1 to WQJ6-1	Stems	Wangqing, Yanbian, Jilin, China	6	WQJ1-1	2018.11	
*S. ginseng*	WQJH1-1 to WQJH1-3	Stems	Wangqing, Yanbian, Jilin, China	3	WQJH1-1	2018.11	
*S. ginseng*	HCG1-1 to HCG9-1	Roots	Hunchun, Yanbian, Jilin, China	9	HCG3-1, HCG5-1	2018.09	
*S. ginseng*	4-1 to 7-1	Roots	Fusong, Baishan, Jilin, China	4	7-1	2019.05	
*S. ginseng*	148-1 to 169-1	Roots	Fusong, Baishan, Jilin, China	21	150-1, 166-1	2019.05	
*S. ginseng*	SJH42-1 to SJH42-4	Roots	Fusong, Baishan, Jilin, China	4	SJH42-2	2019.06	
*S. ginseng*	SJH43-1 to SJH49-1	Roots	Fusong, Baishan, Jilin, China	7	SJH49-1	2019.09	
*S. ginseng*	SJH52-1 to SJH55-1	Roots	Fusong, Baishan, Jilin, China	4		2019.09	
*S. ginseng*	SJH43-2 to SJH50-2	Roots	Fusong, Baishan, Jilin, China	8		2019.09	
*S. ginseng*	LQG1-1 to LQG1-5	Roots	Jingyu, Baishan, Jilin, China	5		2019.09	
*S. ginseng*	LQJ1-1 to LQJ4-1	Stems	Jingyu, Baishan, Jilin, China	4	LQJ2-1	2019.09	
*S. ginseng*	196-1 to 204-1	Roots	Fusong, Baishan, Jilin, China	9	196-1	2020.10	

*S. ginseng*	QYJ4-1 to QYJ4-3	Stems	Fusong, Baishan, Jilin, China	3	QYJ4-1	2020.06	
*S. ginseng*	QYJ5-1 to QYJ5-3	Stems	Fusong, Baishan, Jilin, China	3		2020.06	
*S. ginseng*	QYJ6-1 to QYJ6-2	Stems	Fusong, Baishan, Jilin, China	2		2020.06	
*S. ginseng*	LHG1-1 to LHG1-4	Roots	Liuhe, Tonghua, Jilin, China	4	LHG1-1	2020.06	
*S. sclerotiorum*	YY3 to YY4	Roots	Jingyue, Changchun, Jilin, China	2	YY3, YY4	2018.03	The mycelia appeared white at the initial stage, radial in shape, and flocculent in the late stage on PDA plates; the back of the colony was a gray-white color. The sclerotia produced after 12 days mostly formed at the edge of the Petri dish and were mouse-dung-like, spherical, or kidney-shaped. Relatively fewer sclerotia were present after 20 days on PDA, with 22–28 sclerotia produced in each 9 cm Petri dish. The sclerotia were larger than those of the other two Sclerotinia species, with an average size of 29.82 ± 1.17 mm ×31.21 ± 1.39 mm. All 27 isolates were identified as *S. sclerotiorum* according to their morphology
*S. sclerotiorum*	JH3–JH5	Roots	Jingyue, Changchun, Jilin, China	3		2018.05	
*S. sclerotiorum*	TH3–TH9	Roots	Tonghua, Tonghua, Jilin, China	7	TH3, TH4, TH5, TH6, TH7, TH8, TH9	2018.05	
*S. sclerotiorum*	FS1 to FS9	Roots	Fusong, Baishan, Jilin, China	9	FS1, FS2, FS3, FS4, FS5, FS6, FS7, FS8	2019.06	
*S. sclerotiorum*	THG1	Roots	Tonghua, Tonghua, Jilin, China	1	THG1	2019.06	
*S. sclerotiorum*	FS15 to FS19	Roots	Fusong, Baishan, Jilin, China	5	FS15, FS16	2020.06	

### Fungal isolation and purification

To isolate the pathogen, the diseased samples were washed in running tap water, dried on sterile filter paper, and surface-sterilized in 70% alcohol for 30 s and 2% sodium hypochlorite for 60 s. The samples were rinsed three times in sterile water and cut into 2 mm × 2 mm sections with a sterile knife. The sections were transferred to potato dextrose agar (PDA) plates and incubated in the dark at 20°C for 2 to 3 days. After the mycelia grew, a single marginal mycelium was transferred to a new PDA plate. All isolates were stored in inclined test tubes at 4°C.

### Morphological characterization

The isolates were placed onto PDA medium under 12 h of alternating day and night conditions and cultured at 20°C for 5 days. The colony characteristics were observed and recorded. The number and size of sclerotia in each plate and distribution were quantified and recorded after formed on PDA plates (29). Each experiment was repeated three times.

### DNA extraction, polymerase chain reaction (PCR), and sequencing

According to the morphological identification results, five representative strains were selected from different colony characteristics, including YC5, 30-1, QYJ2-1, KD616, and CBJ2 for type 1; TH4, TH6, TH7, FS2, and FS5 for type 2, and TH1, TH2, SJH42-2, 150-1, and WQJ1-1 for type 3. Each morphotype was chosen and cultured on PDA plates for 3 days. Next, mycelial disks (8 mm) of *Sclerotinia* spp. were inoculated onto PDA plates using a cork borer (8 mm diameter). The mycelia were isolated after 7 days of culture at 25°C under 12 h of alternating day and night. These strains were selected for DNA extraction. The genomic DNA of each isolate was extracted using a Biospin Fungal Genome Extraction Kit (Bioer Technology Co., Ltd., Hangzhou, P. R. China).

The PCR system included 12.5 Microliter (μL) of PCR Premix Taq (TaKaRa Taq Version 2.0), which contained 0.75 U of TaKaRa Taq DNA polymerase, 0.4 millimoles per liter (mM) of dNTP mixture, 20 mM of Tris-HCl, 100 mM of KCl, and 3 mM of MgCl_2_, and then 0.5 µL of the forward and reverse primers (10 µmol·L^−1^), 0.5 µL of genomic DNA, and 10 to 25 µL of ddH_2_O. The cycling parameters for the ITS (30) and the *beta-tubulin* gene (30) consisted of an initial denaturation step at 95°C for 5 min, followed by 34 cycles at 95°C for 30 s, 53°C for 30 s, 72°C for 1 min, and a final cycle at 72°C for 10 min. The amplified products were sequenced (sequencing primers were the same as PCR) by Sangon Biotech Co., Ltd. (Shanghai, China).

The sequences were edited and assembled using DNAMAN software (v9.0; Lynnon Biosoft) to produce consensus sequences and for comparison by BLAST against the NCBI nonredundant database (31). The sequences were submitted to GenBank and assigned accession numbers (Table 2).

**TABLE 2:** Sequences of *Sclerotinia* spp. and GenBank accession numbers used in this study^*a*^

Species	Isolate code	Host	GenBank accession no.	
			ITS	TUB2
*Sclerotinia sclerotiorum*	HP-1	*Nicotiana tabacum*	MK656936	MN295984
*S. sclerotiorum*	HP-2	*N. tabacum*	MK674259	MN295985
*S. sclerotiorum*	GNIY1	*Canavalia ensiformis*	MN163120	MN163119
*S. sclerotiorum*	HMCS-9	*Cardamine hirsuta*	MK216023	MK216022
*S. sclerotiorum*	GPSS003	*Pisum sativum*	MG931017	MG931018
*S. sclerotiorum*	FSD001	*Solanum melongena*	MH457168	MH469747
***S. sclerotiorum***	**TH4**	***Panax ginseng***	**MW375458**	**ON022989**
***S. sclerotiorum***	**TH7**	***P. ginseng***	**MW375455**	**ON022991**
***S. sclerotiorum***	**TH9**	***P. ginseng***	**MW375454**	**ON022990**
***S. sclerotiorum***	**FS2**	***P. ginseng***	**MW375457**	**ON022993**
***S. sclerotiorum***	**FS5**	***P. ginseng***	**MW375456**	**ON022992**
*S. nivalis*	ZJ12	*Actinidia arguta*	KT003216	KT023309
*S. nivalis*	ZJ11	*A. arguta*	KT003215	KT023308
*S. nivalis*	SN2	*P. ginseng*	MW927135	MW929180
*S. nivalis*	SN1	*P. ginseng*	MW927134	MW929179
*S. nivalis*	KGC-S0601	*P. ginseng*	JX262268	JX296007
***S. nivalis***	**CBJ2**	***P. ginseng***	**MW692801**	**ON022986**
***S. nivalis***	**KD616**	***P. ginseng***	**MW692800**	**ON022984**
***S. nivalis***	**QYJ2-1**	***P. ginseng***	**MW692802**	**ON022985**
***S. nivalis***	**YC5**	***P. ginseng***	**MW692803**	**ON022988**
***S. nivalis***	**30-1**	***P. ginseng***	**MT822774**	**ON022987**
*S. minor*	W175	*Arachis hypogaea*	KY701276	KY701265
*S. minor*	F3	*A. hypogaea*	KY701274	KY701264
*S. minor*	W110	*A. hypogaea*	KY701275	KY701263
*S. minor*	E1750	*A. hypogaea*	KY701277	KY701261
*S. cepivorum*	CBS271.30	*Typhula phacorrhiza*	FJ231399	–^*b*^
*S. cepivorum*	CBS189.82	*T. phacorrhiza*	FJ231398	–
*S. cepivorum*	CBS276.93	*T. phacorrhiza*	FJ231400	–
*S. cepivorum*	CBS320.65	*T. phacorrhiza*	FJ231401	–
*S. trifoliorum*	CBS271.30	*Trifolium ambiguum*	JQ743329	–
*S. trifoliorum*	TN Sc4	*Trigonella foenum-graecum*	KT750141	–
*S. trifoliorum*	05WM6	*Cicer arietinum*	EU082464	–
*S. borealis*	SB	*Poa pratensis*	AF067644	–
*S. borealis*	MAFF 241367	*Dumontinia tuberosa*	AB516660	–
*S. homoeocarpa*	PFH0606	*Clarireedia homoeocarpa*	KF725728	KF725741
*S. homoeocarpa*	PFH0480	*C. homoeocarpa*	KF725727	KF725740
*S. homoeocarpa*	PFH0421	*C. homoeocarpa*	KF725726	KF725739
*S. homoeocarpa*	PFH0420	*C. homoeocarpa*	KF725738	KF725738
*S. tetraspora*	2006.1	*Rubus chamaemorus*	Z99672	–
*S. tetraspora*	1993.1	*R. chamaemorus*	Z99671	–
*S. glacialis*	1128.P	*Ranunculus glacialis*	Z99669	–
*S. sativa*	CBS 339.47	*Cannabis sativa*	MH856278	–
*Ciboria americana*	CBS 117.24	*Turfgrass*	MH854767	KF545194
***S. ginseng***	**TH1**	***P. ginseng***	**OM995901**	**ON022994**
***S. ginseng***	**TH2**	***P. ginseng***	**OM995902**	**ON022995**
***S. ginseng***	**SJH42-2**	***P. ginseng***	**OM995905**	**ON022996**
***S. ginseng***	**WQJ1-1**	***P. ginseng***	**OM995899**	**ON022998**
***S. ginseng***	**150-1**	***P. ginseng***	**OM995903**	**ON022997**

^*a*^ The strains in bold are the ones isolated in this study.

^*b*^ – indicates that the relevant sequences were submitted to NCBI by the authors.

### Phylogenetic analysis

One to six strains of the ITS and beta-tubulin (*b-tub*) sequences were selected from *S. sclerotiorum*, *S. nivalis*, *S. minor*, *S. cepivorum*, *S. trifoliorum*, *S. borealis*, *S. tetraspora*, *S. glacialis*, and *S. sativa* from GenBank were randomly selected to construct a multi-locus phylogenetic tree. Their accession numbers are also listed in Table 2. CLUSTALW was used for sequence alignment. DNAMAN (version 9.0) was used to produce consensus sequences. A maximum likelihood phylogenetic tree based on concatenated ITS and *beta-tubulin* sequences of the three phylogenetic analyses was implemented using MEGA 11.0 (32), based on consensus parsimony analyses of concatenated sequences of two ITS and *b-tub* regions for isolates in this study, plus nine related sequences of various *Sclerotinia* spp. obtained from the NCBI (Table 2). *Ciboria americana* (MH854767) was used as the outgroup.

### *In vitro* pathogenicity testing

We selected 60 strains based on morphological and geographically widespread, which represented over 80% of the sampling regions from the purified isolates to inoculate the ginseng roots (Variety: Fu Xing No. 1) *in vitro*. The 3-year-old roots were rinsed thoroughly with sterile water, soaked in 75% ethanol for 1 min, then rinsed with sterile water three times, and blow-dried in a sterile environment. The roots were placed in a sterilized dish covered with sterile paper, and about 20 mL of sterile water was sprayed on the paper to keep it moist. Representative isolates were cultured on PDA at 25°C for 3 days (*S. sclerotiorum*) or 5 days (*S. nivalis* and *S. ginseng*). Mycelial disks were taken from the edge of the colony with 8 mm disks while it was growing to 80% of the diameter of the Petri dish. The disks were placed on the ginseng root, with the mycelium positioned face down without any wounding treatment applied. All ginseng roots were inoculated with two mycelial disks, and about 300 µL sterile water was sprayed on the ginseng root. Finally, the plates were sealed with a sterile plastic lid and inoculated at 25°C for 10 days. A PDA disk served as the non-inoculated control. Five ginseng root replicates were inoculated with each isolate. All tests were repeated three times. The inoculation method for stems and leaves was the same as for roots. The samples were observed every 2 days. Pathogenicity was scored using a 0 to 9 scale, according to the standard of disease occurrence degree by Elshahawy et al. (33). The grading criteria were *level 0*: healthy, no visible lesions; *level 1*: appearance is still healthy, with only a small amount of the white mycelium covering the surface of the ginseng roots; *level 3*: one or two water-soaked lesions with a diameter <2 cm, with the internal tissue around the affected site starting to soften; *level 5*: more than two water-soaked lesions present, or at least a single lesion >2 cm in diameter, with tissues around the affected site soft and loose, and no more than 30% of root area rotten; *level 7*: 30%–50% of the rotting area softened, and the white mycelium on the surface assumed a spherical shape; *level 9*: soft rotted area of >50%, with many mycelia visibly present and black sclerotia forming on the root surface.

### Pathogenicity testing in the greenhouse

The representative isolates used for *in vitro* pathogenicity testing were cultured on PDA plates for growth to 80% of the diameter of the Petri dish, after which the mycelial disks (8 mm in diameter) were placed on potato dextrose medium (PDA medium without agar), and incubated on a shaker (25℃, 150 rpm) (34). Potted ginseng plants were in the full leaf expansion stage (45 d after the cultivation of 4-year-old ginseng) before inoculation. The soil obtained from the non-cultivated ginseng field was sterilized before cultivation. A fungal mycelial suspension of 0.1 g·mL^−1^ was prepared by blending and poured onto the ginseng rhizosphere soil, and 50 mL mycelial suspension was poured into each pot with one ginseng. Ten ginseng plants were inoculated using each isolate. Sterile PDA disks have been used in the liquid culture for the non-inoculated control plants. The pathogenicity of the isolate and symptoms of the aboveground parts after root infection were observed and recorded 14 days later. Disease severity was scored using the same scale as for the *in vitro* pathogenicity testing. Fungi were re-isolated when inoculated plants displayed symptoms like those under natural conditions. They were then identified by ITS sequence analysis.

### Antagonistic effects of *B. amyloliquefaciens* FS6 on *Sclerotinia* species

Three representative strains (*S. nivalis* YC5, *S. sclerotiorum* TH6, and *S. ginseng* 150-1) were selected for each of the three *Sclerotinia* species. The fermentation conditions for *B. amyloliquefaciens* strain FS6 (CGMCC No. 9538) followed those of Wang et al. (27). The concentration of FS6 fermentation broth (FB) was 1 × 10^9^ CFU·mL^−1^. *Sclerotinia* hyphae (8 mm in diameter) were placed at the center of the plate, and a sterile filter paper disk (4 mm in diameter) was positioned at the middle of the left and right sides. Then, 5 µL of FS6 FB was added to the filter paper on the left, and 5 µL of the aseptic FB medium was added on the right side as a control (Fig. 5A). All samples were repeated in triplicate. After culturing at 25°C for 5 days, the radius of the colony was measured, and the mycelial growth inhibitory rate was calculated to evaluate the inhibitory effect of FS6 FB on mycelial growth of *Sclerotinia*. After 5 days of culture, the hyphae were picked from the edge of the inhibition zone, and their morphology was compared to that of normal hyphae under a Zeiss optical microscope. The inhibitory effect of FS6 FB on sclerotia formation was observed after 20 days. The inhibitory rate (%) =100 × (number of sclerotia produced in the control − number of sclerotia produced in the treatment)/number of sclerotia produced in the control.

### Effect of *B. amyloliquefaciens* FS6 fermentation broth on germination of sclerotia

Sclerotia of the three representative strains were collected after 20 days of culture on PDA plates, immersed for 1 min in 75% alcohol to sterilize the hyphae on the surface of the sclerotia, and rinsed three times with sterile water. The FS6 FB was prepared at an initial concentration of 10^9^ CFU·mL^−1^ and diluted 10-, 50-, 100-, 150-, and 250-fold (35), yielding corresponding cell concentrations of 1 × 10^8^, 2 × 10^7^, 1 × 10^7^, 6.67 × 10^6^, and 4 × 10^6^ CFU·mL^−1^. Then, 10 µL of each of the diluted FS6 FB concentrations was added to a PDA plate and coated evenly on the surface of the culture medium with a sterile spreader. Ten sclerotia were placed in each dish, and FS6-free FB of the same volume was used as the control. Each treatment was repeated three times. The number of germinated sclerotia in a given treatment was counted after the germination rate of the control sclerotia exceeded 90% (2 to 3 days).

### Control efficacy of *B. amyloliquefaciens* FS6 FB on *Sclerotinia* species *in vitro*

Roots of similarly sized 4-year-old ginseng with no mechanical damage or wounds were selected for the following tests. The ginseng roots were disinfected in 75% ethanol for 1 min, washed three times in aseptic distilled water, and air-dried. FS6 FB at an initial concentration of 10^9^ CFU·mL^−1^ was diluted to cell concentrations of 1 × 10^8^, 2 × 10^7^, 1 × 10^7^, 6.67 × 10^6^, and 4 × 10^6^ CFU·mL^−1^. The ginseng roots were separately soaked for 10 min in these FS6 FB concentrations and dried. Three representative strains of each *Sclerotinia* species were selected to inoculate mycelial disks (8 mm) on ginseng roots *in vitro*. Ginseng soaked in aseptic FB and inoculated with a mycelial disk was used as the positive control (6, 8). Ginseng inoculated with an aseptic agar disk was used as the negative control. Five ginseng roots were inoculated in each treatment, and all treatments were repeated three times. All treated ginseng roots were placed on a sterilized porcelain plate covered with sterile filter paper and cultured at 25°C. Following the method of Elshahawy et al. (36), the disease level and the number of diseased roots were recorded 15 days post-inoculation (DPI).

The grading criteria were the same as those used for *in vitro* pathogenicity testing.

The disease index and control efficacies were calculated as follows:

Disease index = 100 × ∑ (number of diseased roots at all levels × disease level)/(total root number × maximum level)

Control efficacy (%) = 100 × (disease index in blank control – disease index in treatment)/disease index in blank control

### Field control efficacy of *B. amyloliquefaciens* FS6 on *Sclerotinia* root rot caused by *S. nivalis*

The experiments were conducted from late May to late June in 2021 and 2022, with air temperatures ranging from 11°C to 30°C, soil moisture levels ranging from 80% to 90%, and the soil type identified as agricultural chernozem. FS6 FB at a concentration of 1 × 10^9^ CFU·mL^−1^ was diluted to cell concentrations of 1 × 10^8^, 2 × 10^7^, 1 × 10^7^, 6.67 × 10^6^, and 4 × 10^6^ CFU·mL^−1^, and the FBs of those concentrations were sprayed onto the leaves and stems of ginseng in Changchun. Each treatment was sprayed on a 5 m^2^ area, and each treatment was sprayed three times with 1,500 mL FS6 FB at 7-day intervals. Amino-oligosaccharide 2% water-dispersing granules (Shandong Heyi Biotechnology Co., Ltd., Shandong, China) diluted 150 times with clean water were sprayed on the ginseng plants as the control. The field plots were randomly distributed, and all trials were set up with three replicates. A mycelial suspension containing 0.1 g·mL^−1^ dry *S. nivalis* YC5 mycelia was infused into the roots 7 days after the third application (DATA), with 50 mL applied per root. Ginseng roots (1 m^2^) were dug out at 20 DATA and graded according to the *in vitro* pathogenicity testing conditions, and the disease index and control efficacy of FS6 FB to sclerotinia root rot disease caused by *S. nivalis* YC5 was calculated.

### Data analysis

Differences (mean ± standard error) (*n* = 3 or 6) are expressed according to Tukey’s honestly significant difference test. A *P*-value < 0.05 was considered significant using SPSS statistical software (SPSS Inc., Chicago, IL, USA).

## RESULTS

### Symptoms of ginseng root rot associated with *Sclerotinia*

**Fig 1:**
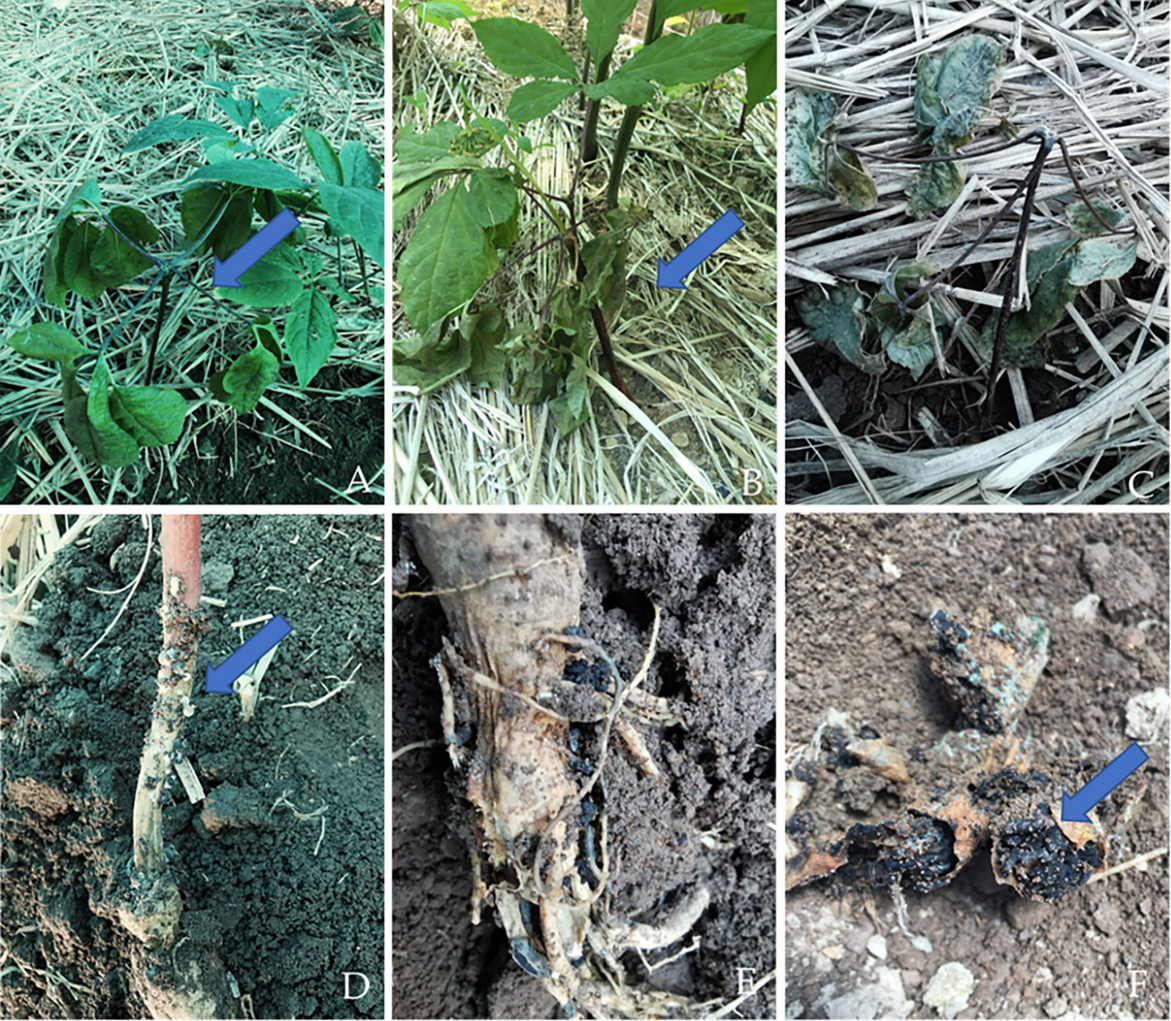
Symptoms of sclerotinia rot in ginseng in fields. (A, B, C) Symptoms in the early, middle, and late stages in the above-ground plant. (D) Symptoms in ginseng stems. (E and F) Symptoms in ginseng roots.

*Sclerotinia* root disease mainly occurs from late April to late September, with two peaks in late April to late June and late August to late September. Sclerotinia disease chiefly harms the root system and stem base of the ginseng plant, and infected roots typically show yellowish water-soaked lesions at the initial stage, after which the root becomes soft, and finally the root surfaces are covered with white mycelia. Numerous black sclerotia form on the surface and inside the ginseng root or the stem, which is accompanied by a distinctive smell. The sclerotia varied in size and shape. The wilting of aboveground leaves was observed during the later stages of root rot, but no significant difference was observed in the symptoms caused by the three different *Sclerotinia* species (Fig. 1).

### Morphological characterization and identification

**Fig 2:**
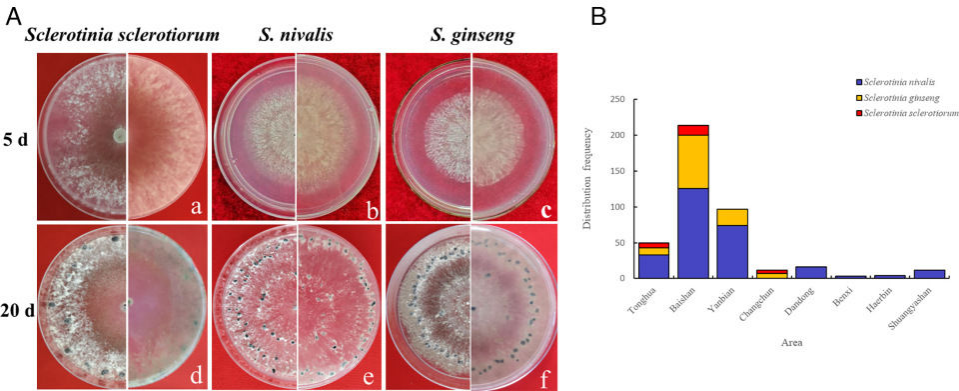
(A) Colony and morphology of representative isolates of Sclerotinia species on PDA for 5 d (a, b, c) and 20 d (d, e, f). (B) Geographical distribution of *Sclerotinia* species in Northeast China.

A total of 409 strains were isolated from 289 symptomatic ginseng root samples collected from 11 planting areas in Jilin, Heilongjiang, and Liaoning Provinces from 2018 to 2021. Based on morphological characteristics (i.e., colony, sclerotia size, and distribution on PDA), the *Sclerotinia* isolates were grouped into three morphotypes. The first morphotype consisted of 27 isolates (6.60%) (YY3, YY4, TH3, TH4, TH5, TH6, TH7, TH8, TH9, FS1, FS2, FS3, FS4, FS5, FS6, THG1, FS7, FS8, FS9, FS15, FS16, FS17, FS18, FS19, JH3, JH4, and JH5; Table 1), whose mycelia grew fastest with an average growth rate of 22.32 ± 0.76 mm·day^−1^. The mycelia appeared white at the initial stage, radial in shape, and flocculent in the late stage on PDA plates (Fig. 2A, a); the back of the colony was a gray-white color (Fig. 2A, d). The sclerotia produced after 12 days mostly formed at the edge of the Petri dish and were mouse-dung-like, spherical, or kidney-shaped. Relatively fewer sclerotia were present after 20 days on PDA, with 22 to 28 sclerotia produced in each 9 cm Petri dish, compared to the other species. The sclerotia were larger than those of the other two *Sclerotinia* species, with an average size of 29.82 ± 1.17 mm ×31.21 ± 1.39 mm. All 27 isolates were identified as *S. sclerotiorum* according to their morphology (Table 1).

The second morphotype consisted of the 268 isolates (65.53%) listed in Table 1. The growth rate of these isolates was moderate, averaging 15.10 mm·day^−1^. The mycelia were white at the initial stage on PDA plates (Fig. 2A, b), dissolving-like and appearing white-yellowish at the late stage. Sclerotia were produced after 15 days and were arranged in two circles in a concentric ring. These sclerotia were spherical, hemispherical, and kidney-shaped and were easily separated from the culture plate (Fig. 2A, e). The number of sclerotia was moderate. After 20 days of culture, 160–180 sclerotia were produced in a 9 cm Petri dish, and their average size was 22.17 ± 1.64 mm ×22.90 ± 1.83 mm. All 268 isolates were identified as *S. nivalis*.

The third strain type consisted of the 114 isolates (27.87%) listed in Table 1. Their growth rates were slow, at 14.00 ± 0.33 mm·day^−1^. The mycelia appeared white and dissolving-like at the earlier stage on PDA plates (Fig. 2A, c) but then turned gray-black during the late stage, and sclerotia were produced after 15–20 days. These sclerotia were arranged in one spherical and hemispherical ring, of which some were irregular in shape and lay close to the surface of the plate (Fig. 2A, f). A moderate number of sclerotia was produced: 132–161 on one 9 cm Petri dish after 20 days of culture on PDA. The sclerotia were medium-sized, averaging 22.53 ± 1.06 mm ×24.63 ± 1.10 mm. All 114 isolates were identified as *S. ginseng* according to their morphology.

*S. nivalis* was the most widely distributed species in all sample-collected regions, whereas *S. ginseng* was isolated only in Jilin Province, and *S. sclerotiorum* was restricted to Tonghua, Baishan, and Changchun cities in Jilin Province (Table 1; Fig. 2B).

### Phylogenetic analysis

The BLASTn searches of the ITS and *beta-tubulin* sequences generated from five purified isolates (YC5, KD616, CBJ2, QYJ2-1, and 30-1) against GenBank showed high similarity (>99%) with the *S. nivalis* sequences, while the other five isolates (FS2, FS6, TH4, TH7, and TH9) showed 98% similarity with the *S. sclerotiorum* sequences. Similarly, the ITS sequences of the other five isolates (SJH42-2, WQJ1-1, 150-1, TH1, and TH2) showed 99% similarity with those of *S. ginseng*.

**Fig 3:**
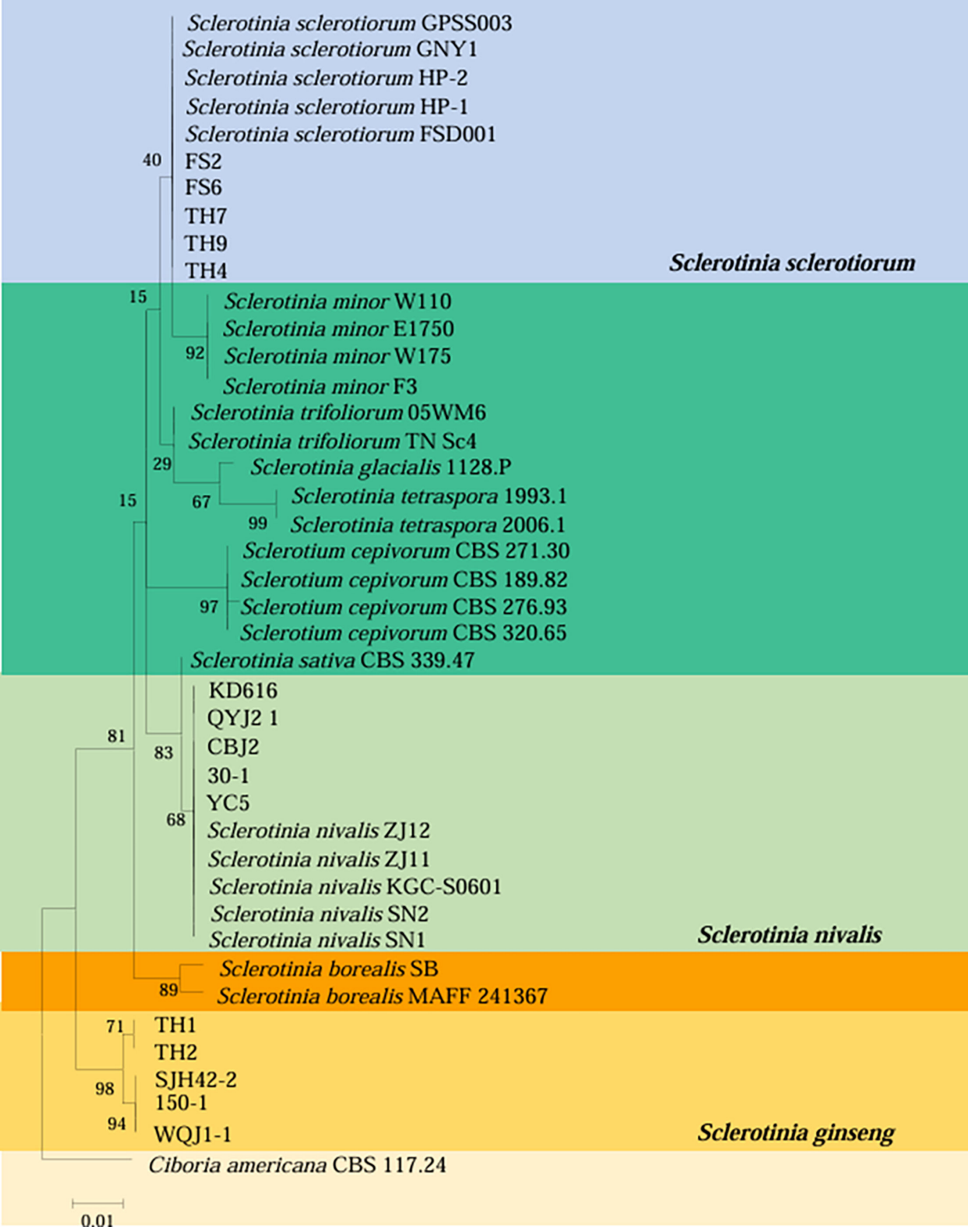
Maximum likelihood phylogenetic tree of *Sclerotinia* spp. based on the sequence analysis of ITS and beta-tubulin genes. The bar represents 0.01 substitutions per nucleotide position.

Five strains (30-1, YC5, KD616, CBJ2, and QYJ2-1) were clustered in *Sclerotinia nivalis* and formed a distinct clade (ML bootstrap support value 99) (Fig. 3). Five isolates (FS2, FS6, TH4, TH7, and TH9) were recognized as *S. sclerotiorum*. Five isolates (SJH42-2, 150-1, WQJ1-1, TH1, and TH2) formed a distinct clade (ML bootstrap support value 99); however, no reference strain was clustered with the five isolates (Fig. 3), which were finally identified as *S. ginseng* according to morphological and molecular characteristics.

Based on the morphology or DNA sequence data, the 268 isolates from Jilin, Liaoning, and Heilongjiang Provinces were identified as *S. nivalis*; the 114 isolates from Jilin Province were identified as *S. ginseng*, and the 27 isolates from Jilin Province were identified as *S. sclerotiorum*; all three representative species were distinct from the other *Sclerotinia* species (Table 2, Fig. 3). The molecular identification results corroborated those based on morphology.

### Pathogenicity tests

**Fig 4:**
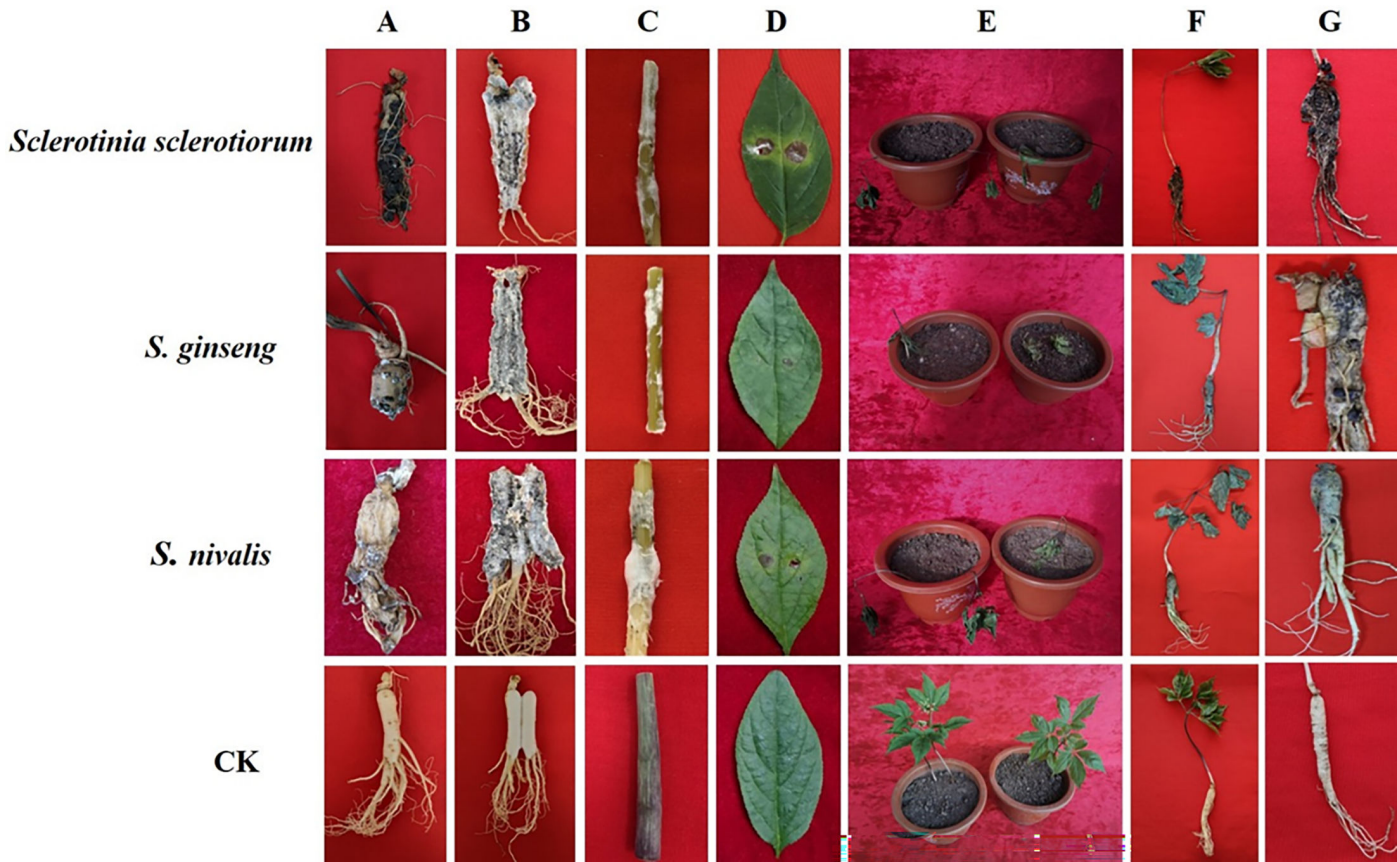
Pathogenic symptoms of different isolates of *Sclerotinia* species on different parts of ginseng. (A) Roots in natural conditions; (B) roots inoculated *in vitro*; (C) stems inoculated *in vitro*; (D) leaves inoculated *in vitro*; and (E through G) whole ginseng plants inoculated *in vivo*.

Water-immersed yellow plaques appeared *in vitro* at 5 DPI, and white flocculent mycelia grew outward from the disease site. After 10 days, the mycelia spread to the entire ginseng root and began to gather into white globules, which is the initial stage of forming sclerotia. The root was sectioned in half, and the internal tissue root revealed evidence of water immersion, soft rot, and a unique smell, with black sclerotia forming inside the root during the late stage (Fig. 4B). The infection ability of the three species was almost the same, and all of the species infected the stem. The stem presented water stains at 3 DPI, with white mycelia spreading outward from the disease site and covering the whole stem. The stem turned soft and rotten at 7 DPI, and the stem infection ability was similar among the three species (Fig. 4C). *Sclerotinia* spp. slightly infected the leaves, leading to limited necrotic spots observed at 7 DPI (Fig. 4D).

In addition, we selected the same 15 isolates to inoculate ginseng roots *in vivo* in the greenhouse. The aboveground leaves lost their green color and wilted, while the belowground part had only a few white cotton-like mycelia, but the internal tissue remained healthy at the initial inoculation stage; the aboveground leaves dried up due to dehydration at 14 DPI. The initial sclerotia that formed from the accumulated mycelia on the surface of the ginseng root were observed below ground once they became soft and odorless, changing in color from light yellow to grayish yellow. At 20 DPI, the aboveground part was devoid of water and had dried, and the ginseng root softened and rotted but with no discernible odor (Fig. 4E). A few black sclerotia were scattered on the surface of the rotten root, and the entire ginseng root turned brown.

However, a large number of sclerotia were found after peeling off the epidermis, and the internal tissue was rotten and had turned brown from outside to inside. Finally, the pulp tissue of the ginseng root was eliminated, leaving only epidermal tissue (Fig. 4F and G). The pathogens were re-isolated from the diseased ginseng, and the isolates were confirmed to be the same *Sclerotinia* species as the original inoculated strains, via both morphological observations and molecular identification. No symptoms were observed, and no *Sclerotinia* spp. were re-isolated from the control; hence, Koch’s postulates were fulfilled.

### Inhibitory effect of *B. amyloliquefaciens* FS6 FB against *Sclerotinia* species

**Fig 5:**
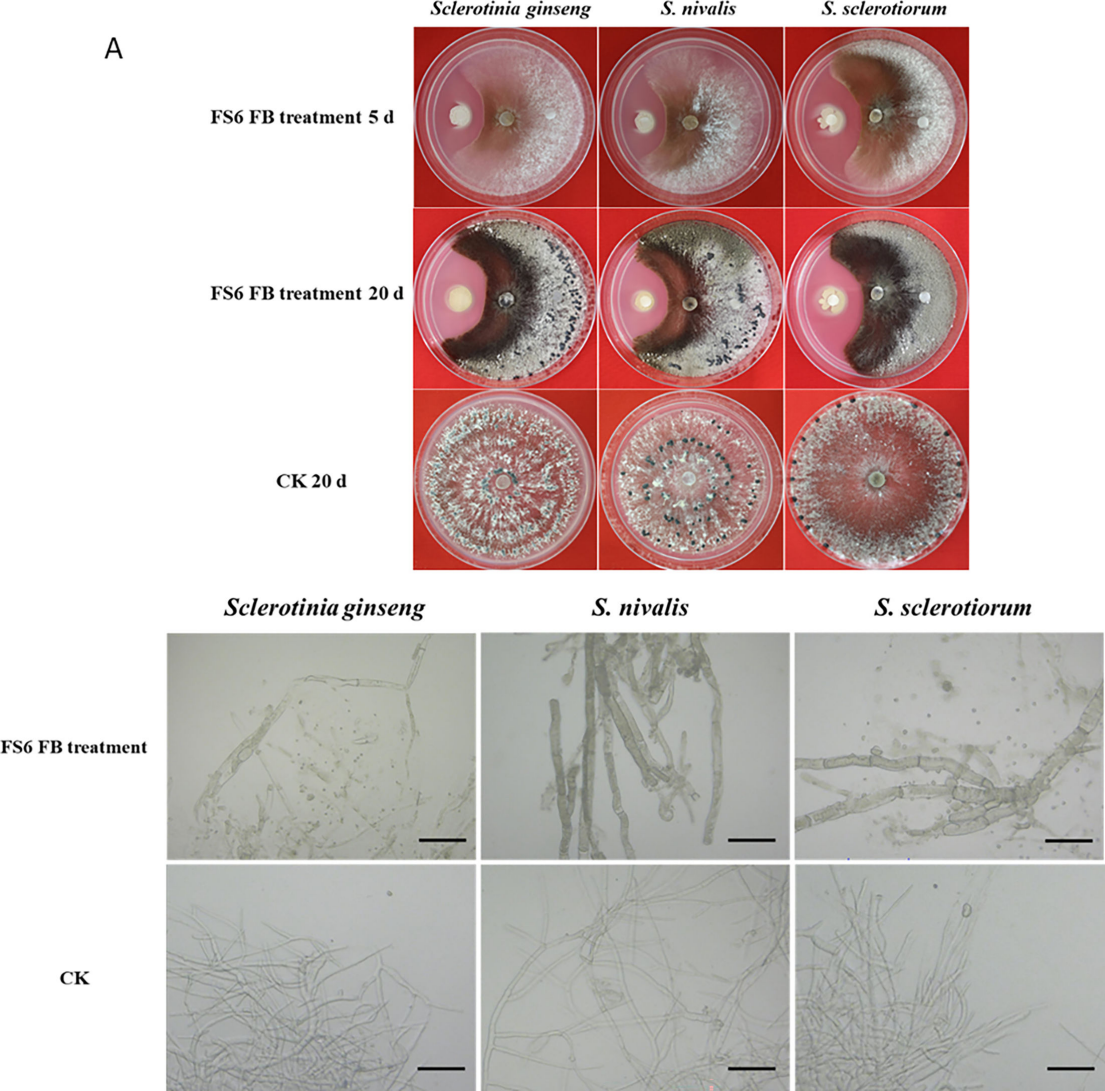
(A) Antagonistic culture of *Bacillus amyloliquefaciens* FS6 fermentation broth (FB) with three species of Sclerotinia for 5 d and 20 d on PDA; (B) antagonistic effect comparisons of FS6 FB on the morphology of mycelia of three species between treatments (upline) and no treatment (downline, CK) under optical microscopy. Bar=75 µm.

The antagonistic culture of *B. amyloliquefaciens* FS6 FB with *Sclerotinia* spp. showed that FS6 FB had a strong inhibitory effect on the growth of mycelia and the formation of sclerotia in the three *Sclerotinia* species (Fig. 5A). Among them, FS6 FB had the strongest inhibitory effect on *S. sclerotiorum*, inhibiting 93.84% of mycelial growth and 88.00% of the sclerotia. The mycelial growth inhibitory rates of FS6 FB for *S. ginseng* and *S. nivalis* were 92.08% and 85.19%, respectively, with sclerotia formation correspondingly inhibited by 73.89% and 75.54%. Microscopic observations revealed that FS6 FB destroyed the mycelia, as characterized by dissolved mycelia, inclusion outflow, thickened hyphal cell walls, a dark color, and uneven protoplast distribution, which contrasted with the untreated normal mycelia (Fig. 5B).

### Effect of *B. amyloliquefaciens* FS6 FB on sclerotia germination in *Sclerotinia* spp

Different concentrations of FS6 FB had pronounced inhibitory effects on the germination of sclerotia. Sclerotia treated with FS6 FB at concentrations of 1 × 10^8^, 5 × 10^7^, 1 × 10^7^, 6.67 × 10^6^, or 4 × 10^6^ CFU·mL^−1^ showed no signs of germination at 3 days post-treatment when the sclerotia germination rate in the control Petri dish exceeded 60%; all sclerotia treated with FS6 failed to germinate under the treatment for at least 20 days (Table 3).

**TABLE 3:** Inhibition effect of *Bacillus amyloliquefaciens* FS6 fermentation broth (FB) on sclerotia germination of *Sclerotinia* spp. on PDA^*a*^

Concentration of FS6 FB CFU mL^−1^	*Sclerotinia nivalis*		*S. ginseng*		*S. sclerotiorum*	
	Germination rate	Inhibition rate %	Germination rate	Inhibition rate %	Germination rate	Inhibition rate %
1 × 10^8^	0.00	100.00 ± 0.00 a	0.00	100.00 ± 0.00 a	0.00	100.00 ± 0.00 a
5 × 10^7^	0.00	100.00 ± 0.00 a	0.00	100.00 ± 0.00 a	0.00	100.00 ± 0.00 a
1 × 10^7^	0.00	100.00 ± 0.00 a	0.00	100.00 ± 0.00 a	0.00	100.00 ± 0.00 a
6.67 × 10^6^	0.00	100.00 ± 0.00 a	0.00	100.00 ± 0.00 a	0.00	100.00 ± 0.00 a
4 × 10^6^	0.00	100.00 ± 0.00 a	0.00	100.00 ± 0.00 a	0.00	100.00 ± 0.00 a
CK	100.00	–^*b*^	100.00	–	100.00	–

^*a*^ Data presented are the means ± SE. The lowercase letters indicate significant difference (*P* < 0.05) among the treatments.

^*b*^ – indicates that the blank control treatment showed no fungal inhibition effect.

### Control efficacy of *B. amyloliquefaciens* FS6 FB on sclerotinia root rot caused by the three *Sclerotinia* species *in vitro*

**Fig 6:**
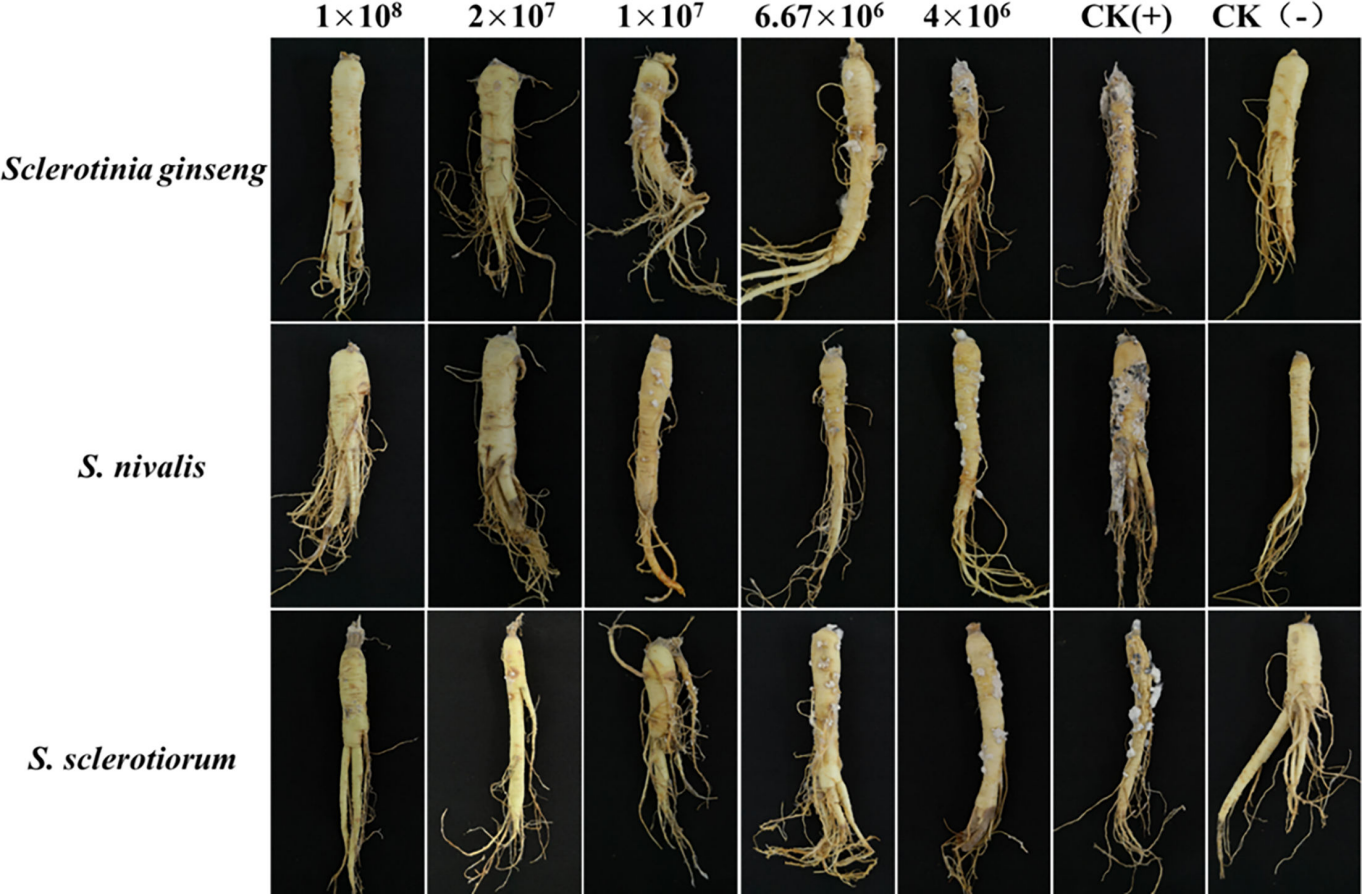
Control efficacies of *Bacillus amyloliquefaciens* FS6 fermentation broth at different concentrations against ginseng *Sclerotinia* disease in greenhouse. The concentration unit for the *B. amyloliquefaciens* FB used in the figure is expressed as CFU/mL.

The ginseng roots treated with FS6 FB did not change at 5 DPI. In contrast, the roots of the control began to develop watery lesions at the inoculation site, and a few mycelia had spread on the ginseng root surface, corresponding to disease level 1. At 15 DPI, the diseased area on the ginseng roots in the control reached more than 80%, but no disease symptoms were detected in the ginseng roots treated with 1 × 10^8^ CFU·mL^−1^ of FS6 FB and inoculated with either *S. ginseng* or *S. nivalis*, for which the control efficacy values were 100%; that of FS6 FB on *S. sclerotiorum* was 98.91%. At a concentration of 6.67 × 10^6^ CFU/mL, *B. amyloliquefaciens* FS6 FB exhibited variable *in vitro* control efficacy against *Sclerotinia* diseases caused by different *Sclerotinia* species. Control efficacy against *S. sclerotiorum*-induced disease was 91.30%, while it was 87.50% against *S. ginseng*-induced disease. However, control efficacy against *S. nivalis*-induced disease was significantly lower at 70.21%. These differences were statistically significant at *P* < 0.05. No significant difference in *in vitro* control efficacy against *Sclerotinia* disease caused by the three *Sclerotinia* species was observed when treated with *B. amyloliquefaciens* FS6 FB at a concentration of 4.0 × 10^6^ CFU/mL (*P* < 0.05) (Table 4, Fig. 6).

**TABLE 4:** Control efficacies of *Bacillus amyloliquefaciens* FS6 fermentation broth (FB) against ginseng *Sclerotinia* disease *in vivo* (2021)^*a*^

Concentration of FS6 FB CFU mL^−1^	*Sclerotinia nivalis*		*S. ginseng*		*S. sclerotiorum*	
	Disease index	Control efficacy %	Disease index	Control efficacy %	Disease index	Control efficacy %
1.00 × 10^8^	0.00	100.00 ± 0.00 a	0.00	100.00 ± 0.00 a	0.93	98.91 ± 0.14 a
5.00 × 10^7^	0.00	100.00 ± 0.00 a	0.93	98.95 ± 0.11b	1.85	97.83 ± 0.27ab
1.00 × 10^7^	5.09	94.15 ± 0.22b	3.15	96.46 ± 0.26 c	2.66	96.88 ± 0.25b
6.67 × 10^6^	25.93	70.21 ± 0.63 c	11.11	87.50 ± 0.53d	7.41	91.30 ± 0.11 c
4.00 × 10^6^	47.22	45.75 ± 1.89d	42.59	52.09 ± 2.01e	48.15	43.48 ± 2.51d
CK	87.04	–^*b*^	88.89	–	85.19	–

^*a*^ Data presented are the means ± SE. The lowercase letters indicate significant difference (*P* < 0.05) among the treatments.

^*b*^ – indicates that the blank control treatment showed no control efficacy *in vivo*.

### Control efficacy of *B. amyloliquefaciens* FS6 FB on sclerotinia root rot caused by *Sclerotinia nivalis* under field conditions

The field assay results revealed that FS6 FB sprayed at 1 × 10^8^, 2 × 10^7^, 1 × 10^7^, 6.67 × 10^6^, and 4 × 10^6^ CFU·mL^−1^ onto ginseng stems and leaves (in Changchun in 2021 and 2022) had good control efficacy for sclerotinia root rot. The efficacy of *B. amyloliquefaciens* FS6 against *S. nivalis* was augmented by increasing the FS6 FB concentration. The efficacy rates of FS6 FB sprayed with 1 × 10^8^ CFU·mL^−1^ in 2021 and 2022 were 71.20% and 73.51%, respectively, which were significantly higher than those obtained with amino-oligosaccharides (49.00% and 48.61%). Field experiments demonstrated a negative correlation between FS6 FB concentration and disease development: higher concentrations of FS6 resulted in delayed symptom expression and reduced disease incidence. In the early stage, no aboveground disease symptoms were apparent, but roots became soft. As the disease progressed, roots developed a soft rot, and aboveground tissues exhibited wilting and dehydration. In the final stages, complete plant wilt occurred, accompanied by root decay and the formation of black sclerotia on the root surface (Table 5).

**TABLE 5:** Induced efficacies of *Bacillus amyloliquefaciens* FS6 fermentation broth (FS6 FB) spraying three times on stems and leaves of ginseng for the control of *Sclerotinia* disease in field (Changchun, 2021 and 2022)^*a*^

Results	Concentration of FS6 FB/ CFU·mL^−1^										Amino-oligosaccharide AS		CK	
	1 × 10^8^		5 × 10^7^		1 × 10^7^		6.67 × 10^6^		4 × 10^6^		2%		–^*b*^	–
	2021	2022	2021	2022	2021	2022	2021	2022	2021	2022	2021	2022	2021	2022
Disease index	26.67	24.82	27.78	27.59	43.52	40.37	74.07	68.15	81.48	79.63	47.22	48.15	92.59	93.7
Efficacy %	71.20 ± 3.31 a*^c^	73.51 ± 0.89 a*	69.80 ± 2.95b	70.55 ± 1.49b	53.00 ± 3.82 c	56.91 ± 0.87 c	20.00 ± 4.03e	27.27 ± 1.20e	12.00 ± 4.65 f	15.01 ± 1.80 f	49.00 ± 2.80d	48.61 ± 1.91d	–	–

^*a*^ Data are presented as means ± SE. The lowercase letters after numbers in the same line indicate significant differences (*P* < 0.05) among the treatments.

^*b*^ – indicates that the blank control treatment showed no control efficacy.

^*c*^ * denotes that the treatment shows statistically significant differences at *P* < 0.05.

## DISCUSSION

Among the pathogens responsible for *Sclerotinia* disease on ginseng, *S. ginseng* was reported on *Panax ginseng* in Korea in 1985 (16), *S. nivalis* was first reported on *P. ginseng* in South Korea in 2013 (9), on *P. quiquefolium* in China (18), and on post-harvested Asian ginseng in China in 2022, while *S. sclerotiorum* was reported as the pathogen causing sclerotinia root rot on *P. quiquefolium* in Canada in 1916 (3), in Korea in 2013 (9), and in China in 2022 (20). Based on morphological evaluations and polygenetic sequence analysis of the ITS and *beta-tubulin* sequences of the *Sclerotinia* strains isolated from 11 *P. ginseng* planting areas in Northeastern China, three *Sclerotinia* species caused sclerotinia disease in Asian ginseng in China, including *S. ginseng*, *S. nivalis,* and *S. sclerotiorum*. This study is the first to report *S. nivalis* as a dominant species causing sclerotinia root rot in Asian ginseng cultivated in China. In addition, *S. nivalis* also causes sclerotinia root rot disease in other plant hosts, such as *Atractylodes macrocephala*, *Pulsatilla adan*, and *Lactuca sativa* (37–39). Disease caused by *S. sclerotiorum* has been reported in a wide range of hosts as this fungus infects many broad-leaved plants, including rape, sunflower, eggplant, and pepper (40–42). It was first reported as the causal agent of sclerotinia root rot disease on Asian ginseng in China.

Sclerotia are the only form of *Sclerotinia* spp. that can survive over winter, and they constitute the primary infection source of sclerotinia disease outbreaks in the field as they can survive in soil for 7–8 years or longer (43). The survival of *Sclerotinia* spp. largely depends on the germination and viability of their sclerotia. Because sclerotia are highly resistant to stress, it is difficult to eliminate them from the soil; instead, reducing the number of sclerotinia that are allowed to form under production is a better approach, which is critical for robust disease control of sclerotinia disease in future ginseng crops. Furthermore, the wide host range of sclerotia renders them particularly challenging to control. Fungicides are the principal tool for controlling sclerotinia root rot (44–47). Although applying chemical fungicides is much more effective than doing nothing (i.e., control), these chemicals have negative environmental impacts, and their efficacy wanes over time (48). Accordingly, the use of microbial agents or strains to prevent and control sclerotinia disease has garnered attention and has become a research focus. The antifungal effects of many biocontrol agents or strains, such as *Pseudomonas fluorescens, Trichoderma harzianum, B. amyloliquefaciens,* and *B. subtilis*, on mycelial growth and sclerotia formation of *S. sclerotiorum* have been reported for many different crops (except ginseng) (49–51). Furthermore, *B. thuringiensis* strains effectively suppress *S. sclerotiorum* growth by inducing systemic resistance in *Brassica campestris* according to challenge-inoculation assays (52, 53). These findings are consistent with the effect of *B. amyloliquefaciens* FS6 on *Sclerotinia* spp. from ginseng in our study. Our findings suggest that *B. amyloliquefaciens* FS6 FB has a strong antagonistic effect by inhibiting mycelial growth and formation of sclerotia in all three *Sclerotinia* species. The mycelia became deformed, coarse, dark, and lost hyphal contents.

More surprisingly, *B. amyloliquefaciens* FS6 FB inhibited mycelial growth and reduced the number of sclerotia but also effectively inhibited its germination. When treated with 1 × 10^8^–4 × 10^6^ CFU·mL^−1^ of FS6 FB, the sclerotia did not germinate for at least 20 days. Further inoculation assays in the greenhouse showed that FS6 FB had control efficacies of 98.91%–100% against the three *Sclerotinia* spp. when 1 × 10^8^ CFU·mL^−1^ was applied to ginseng roots. Hence, *B. amyloliquefaciens* FS6 limited sclerotinia disease afflicting ginseng by inhibiting mycelial growth and the formation and germination of sclerotia.

In this study, foliar application of FS6 FB demonstrated sustained control efficacy against ginseng Sclerotinia rot for up to 15 days. No investigations were conducted on its efficacy beyond this period; relevant studies should be further carried out. Previous studies revealed that root irrigation with FS6 FB enabled its survival in soil for over 60 days, with soil microbial communities reaching equilibrium states after this duration (26). Current findings indicate that foliar treatment with FS6 FB significantly modifies the rhizosphere soil microbial community structure of ginseng plants and actively recruits beneficial microorganisms.

Based on 2 years of continuous monitoring, we have not observed any evidence of *Sclerotinia* spp. developing resistance to *B. amyloliquefaciens* FS6. We will continue to monitor for the potential development of resistance to FS6 by *Sclerotinia* spp. under both laboratory and field conditions.

Reglinski proposed that salicylic acid and 4-chlorosalicylic acid induce host resistance mechanisms, and this induced resistance could provide practical control of foliar diseases in horticultural crops (54). Secondary metabolites or antifungal substances derived from *B. amyloliquefaciens* could be used to induce plants to develop resistance or gene expression-related resistance against *S. sclerotiorum* (55–59). FS6 also stimulated Induced Systematic Resistance (ISR) to *S. nivalis* on ginseng, such that the induction efficacy of FS6 FB after spraying with 1 × 10^8^ CFU·mL^−1^ peaked at 71.20% and 73.51%, respectively, 20 days post-treatment in 2021 and 2022. Therefore, combining previous research in our laboratory (26, 28) and this study, *B. amyloliquefaciens* FS6 engaged in direct and indirect modes of action as a potential biocontrol agent. It was directly antagonistic against *Sclerotinia* spp. and indirectly induced resistance to ginseng against sclerotinia root rot disease, as inferred from the field and greenhouse experiments. Nonetheless, the duration and mechanism of FS6-mediated induced resistance to sclerotinia root rot on ginseng need further detailed study.

Future research is needed to fully understand the mechanisms and duration of *B. amyloliquefaciens* FS6-mediated induced resistance (ISR) against Sclerotinia root rot in ginseng, and to optimize its formulation and application strategies for consistent and long-lasting disease control while minimizing the risk of resistance development.

### Conclusion

The strains isolated from Sclerotinia root rot of *Panax ginseng* in northeast China were identified as three species: *Sclerotinia nivalis*, *S. ginseng*, and *S. sclerotiorum*, with *S. nivalis* being the dominant species (accounting for isolation frequency of 65.53%), followed by *S. sclerotiorum* (27.87%) being found on *Panax ginseng* for the first time in China. *B. amyloliquefaciens* FS6 exhibited significant antifungal activity against *Sclerotinia* species. It demonstrated effective inhibition on mycelial growth, sclerotia formation, and sclerotia germination of all three pathogens. Foliar spray application of FS6 FB induced systemic resistance against Sclerotinia rot in ginseng roots, achieving a maximum control efficacy of 73.51% after 15 days. This study reveals that FS6 can effectively control ginseng Sclerotinia rot through convenient foliar spraying, which offers simpler operation compared to root irrigation methods, demonstrating substantial potential for managing this disease.

## SUPPLEMENTAL MATERIAL

Figure S1 spectrum.00470-25-s0001.tiff Antagonistic culture of *Bacillus amyloliquefaciens* FS6 fermentation broth (FB) with three species of Sclerotinia for 10 d on PDA.

ASM does not own the copyrights to Supplemental Material that may be linked to, or accessed through, an article. The authors have granted ASM a non-exclusive, world-wide license to publish the Supplemental Material files. Please contact the corresponding author directly for reuse.
