# Supplementary figures and images for: Sclerotinia spp. causing root rot of Panax ginseng in Northeast China and its potential biocontrol by Bacillus amyloliquefaciens FS6
Source: Microbiol Spectr. 2025 Sep 3;13(10):e00470-25. doi: 10.1128/spectrum.00470-25 (PMC12502780; doi:10.1128/spectrum.00470-25)

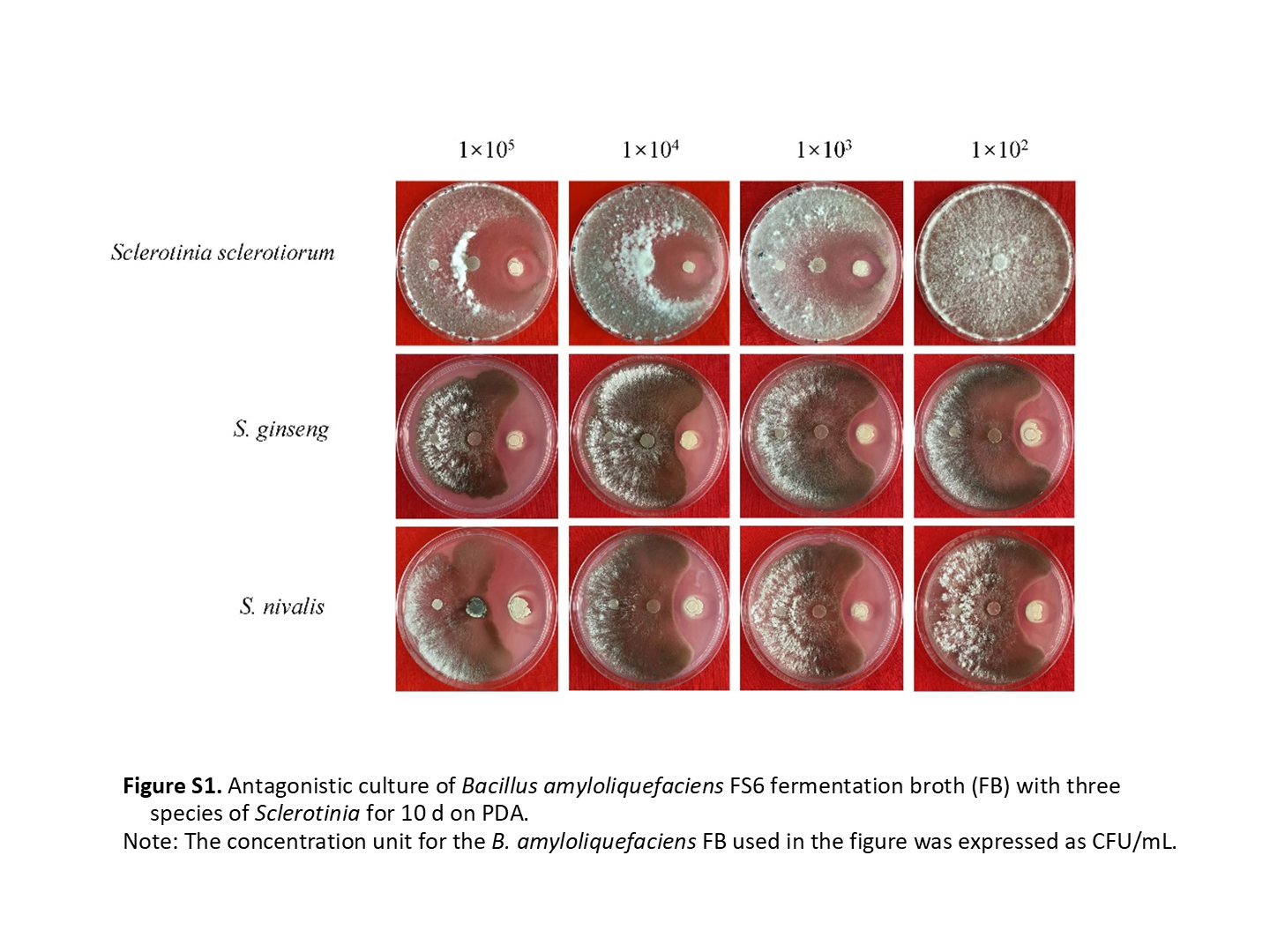

Supplement: Figure S1 — Antagonistic culture of Bacillus amyloliquefaciens FS6 fermentation broth (FB) with three species of Sclerotinia for 10 d on PDA. [file spectrum.00470-25-s0001.tiff]
